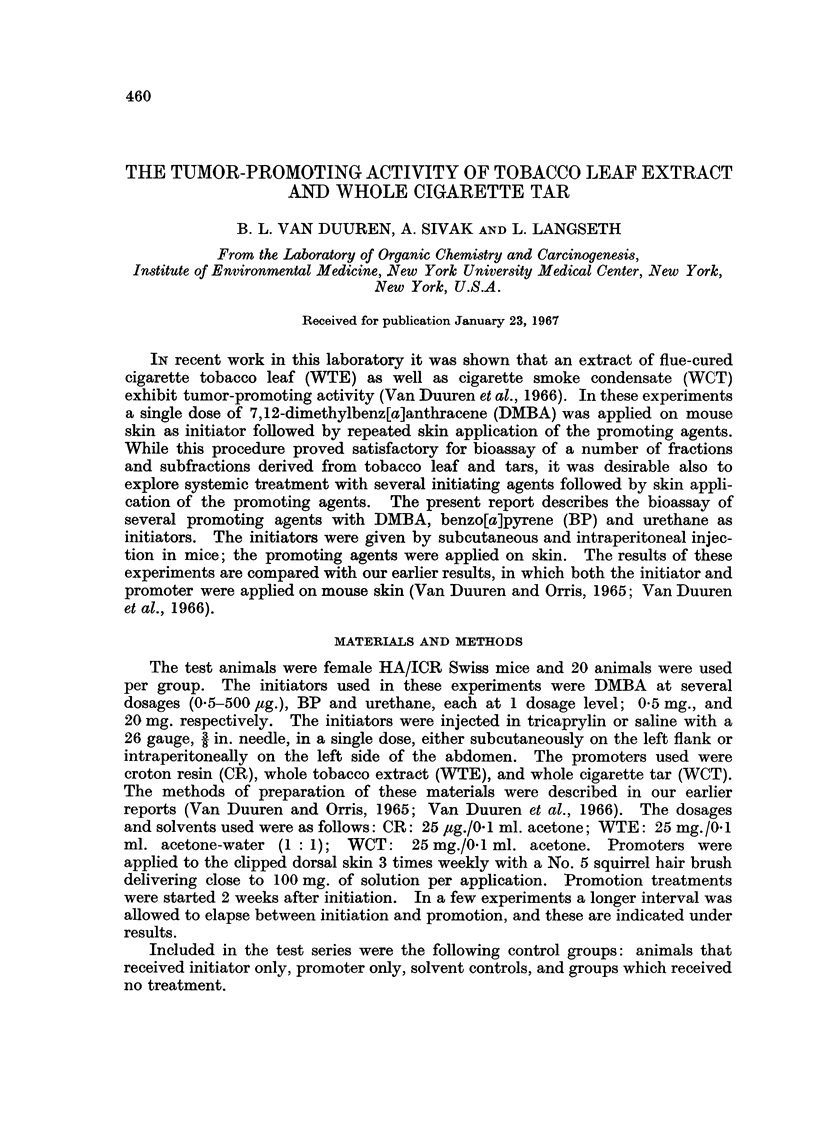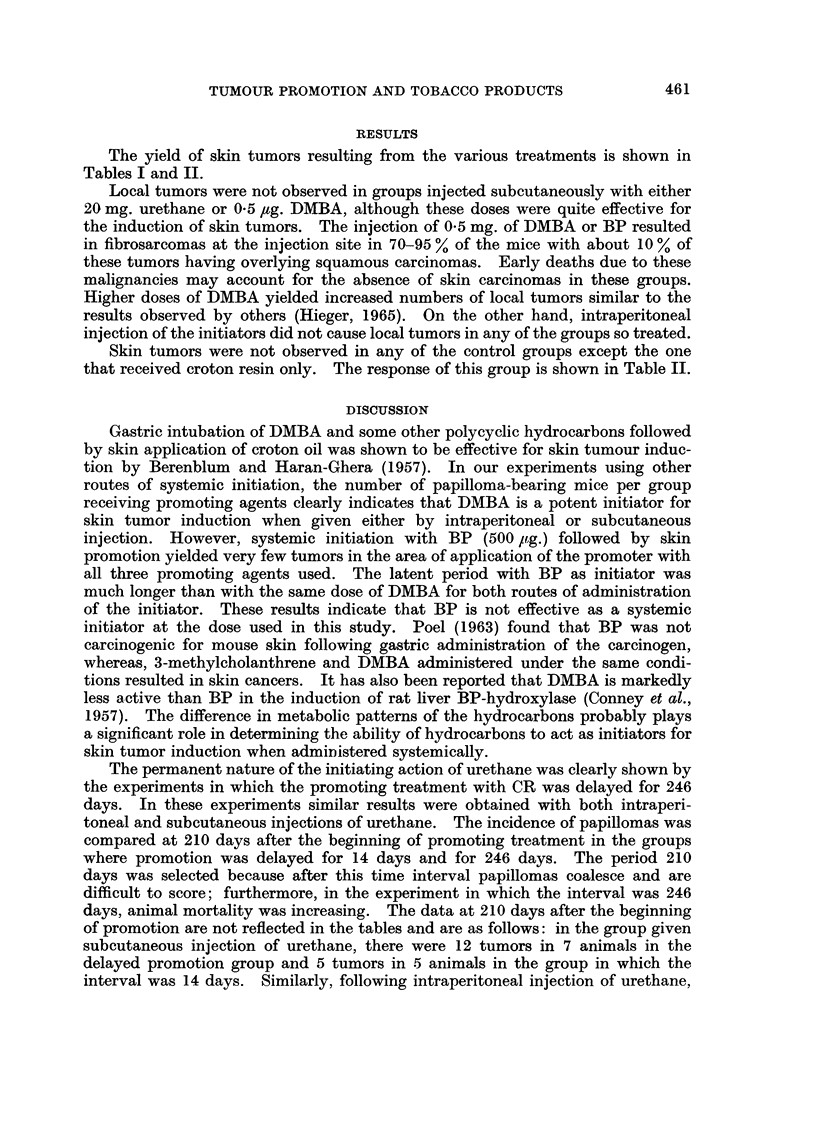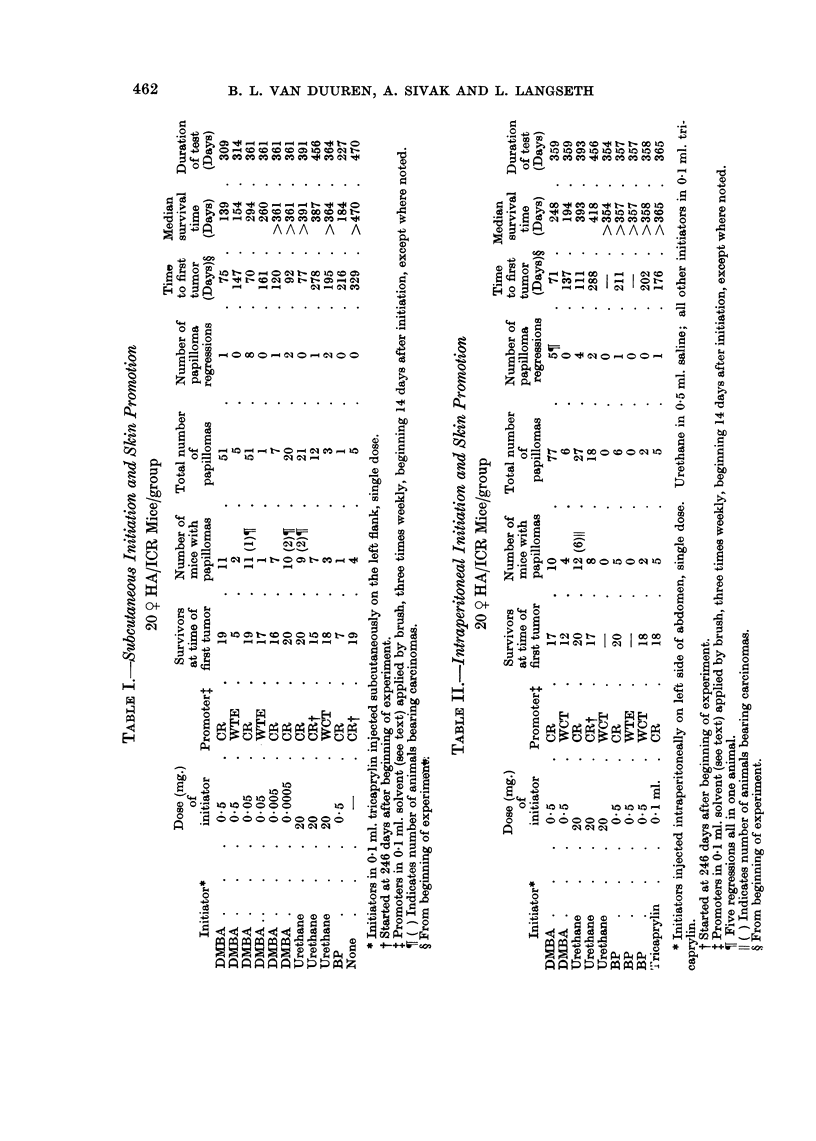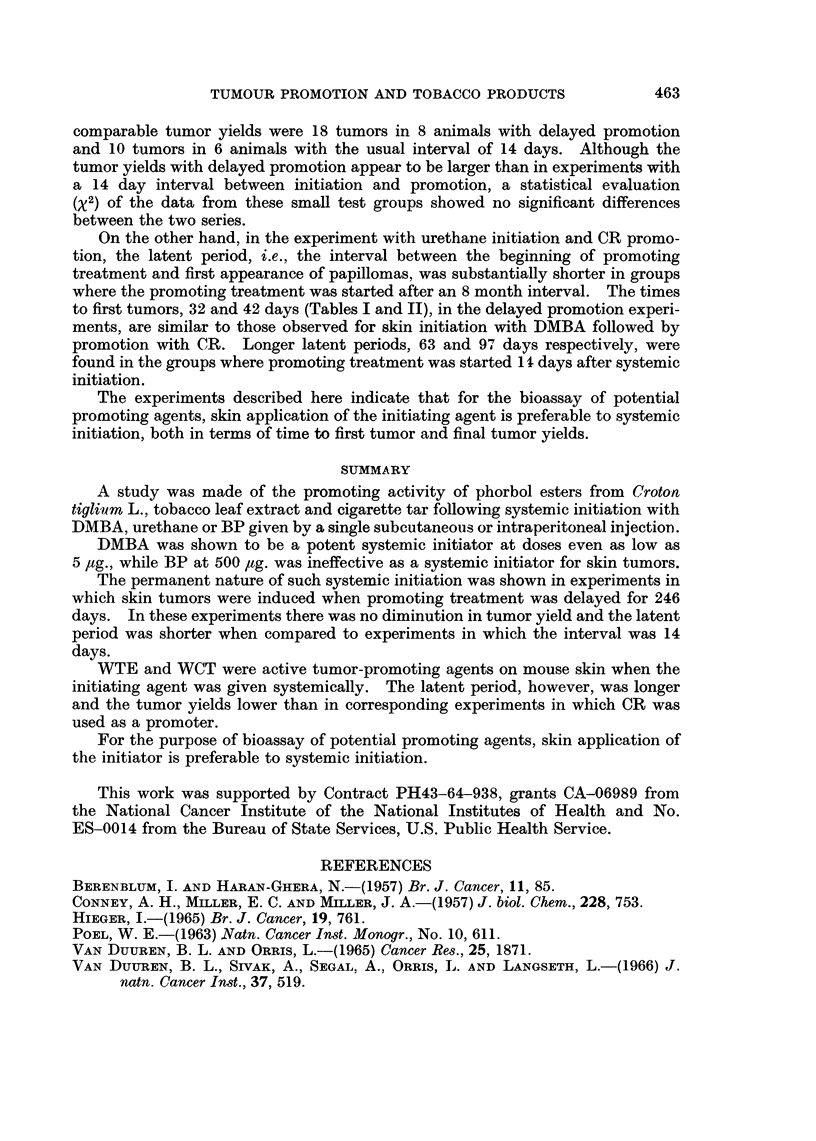# The tumor-promoting activity of tobacco leaf extract and whole cigarette tar.

**DOI:** 10.1038/bjc.1967.53

**Published:** 1967-06

**Authors:** B. L. Van Duuren, A. Sivak, L. Langseth


### THE TUMOR-PROMOTING ACTIVITY OF TOBACCO LEAF EXTRACT AND WHOLE CIGARETTE TAR

B. L. VAN DUUREN, A. SIVAK AND L. LANGSETH

*From the Laboratory of Organic Chemistry and Carcinogenesis, Institute of Environmental Medicine, New York University Medical Center, New York, New York, U.S.A.*

Received for publication January 23, 1967

IN recent work in this laboratory it was shown that an extract of flue-cured cigarette tobacco leaf (WTE) as well as cigarette smoke condensate (WCT) exhibit tumor-promoting activity (Van Duuren *et al.*, 1966). In these experiments a single dose of 7,12-dimethylbenz[*a*]anthracene (DMBA) was applied on mouse skin as initiator followed by repeated skin application of the promoting agents. While this procedure proved satisfactory for bioassay of a number of fractions and subfractions derived from tobacco leaf and tars, it was desirable also to explore systemic treatment with several initiating agents followed by skin application of the promoting agents. The present report describes the bioassay of several promoting agents with DMBA, benzo[*a*]pyrene (BP) and urethane as initiators. The initiators were given by subcutaneous and intraperitoneal injection in mice; the promoting agents were applied on skin. The results of these experiments are compared with our earlier results, in which both the initiator and promoter were applied on mouse skin (Van Duuren and Orris, 1965; Van Duuren *et al.*, 1966).

### MATERIALS AND METHODS

The test animals were female HA/ICR Swiss mice and 20 animals were used per group. The initiators used in these experiments were DMBA at several dosages (0·5–500 $\mu$g.), BP and urethane, each at 1 dosage level; 0·5 mg., and 20 mg. respectively. The initiators were injected in tricaprylin or saline with a 26 gauge, $\frac{5}{8}$ in. needle, in a single dose, either subcutaneously on the left flank or intraperitoneally on the left side of the abdomen. The promoters used were croton resin (CR), whole tobacco extract (WTE), and whole cigarette tar (WCT). The methods of preparation of these materials were described in our earlier reports (Van Duuren and Orris, 1965; Van Duuren *et al.*, 1966). The dosages and solvents used were as follows: CR: 25 $\mu$g./0·1 ml. acetone; WTE: 25 mg./0·1 ml. acetone-water (1 : 1); WCT: 25 mg./0·1 ml. acetone. Promoters were applied to the clipped dorsal skin 3 times weekly with a No. 5 squirrel hair brush delivering close to 100 mg. of solution per application. Promotion treatments were started 2 weeks after initiation. In a few experiments a longer interval was allowed to elapse between initiation and promotion, and these are indicated under results.

Included in the test series were the following control groups: animals that received initiator only, promoter only, solvent controls, and groups which received no treatment.

### RESULTS

The yield of skin tumors resulting from the various treatments is shown in Tables I and II.

Local tumors were not observed in groups injected subcutaneously with either 20 mg. urethane or 0·5 $\mu$g. DMBA, although these doses were quite effective for the induction of skin tumors. The injection of 0·5 mg. of DMBA or BP resulted in fibrosarcomas at the injection site in 70–95 % of the mice with about 10 % of these tumors having overlying squamous carcinomas. Early deaths due to these malignancies may account for the absence of skin carcinomas in these groups. Higher doses of DMBA yielded increased numbers of local tumors similar to the results observed by others (Hieger, 1965). On the other hand, intraperitoneal injection of the initiators did not cause local tumors in any of the groups so treated.

Skin tumors were not observed in any of the control groups except the one that received croton resin only. The response of this group is shown in Table II.

### DISCUSSION

Gastric intubation of DMBA and some other polycyclic hydrocarbons followed by skin application of croton oil was shown to be effective for skin tumour induction by Berenblum and Haran-Ghera (1957). In our experiments using other routes of systemic initiation, the number of papilloma-bearing mice per group receiving promoting agents clearly indicates that DMBA is a potent initiator for skin tumor induction when given either by intraperitoneal or subcutaneous injection. However, systemic initiation with BP (500 $\mu$g.) followed by skin promotion yielded very few tumors in the area of application of the promoter with all three promoting agents used. The latent period with BP as initiator was much longer than with the same dose of DMBA for both routes of administration of the initiator. These results indicate that BP is not effective as a systemic initiator at the dose used in this study. Poel (1963) found that BP was not carcinogenic for mouse skin following gastric administration of the carcinogen, whereas, 3-methylcholanthrene and DMBA administered under the same conditions resulted in skin cancers. It has also been reported that DMBA is markedly less active than BP in the induction of rat liver BP-hydroxylase (Conney *et al.*, 1957). The difference in metabolic patterns of the hydrocarbons probably plays a significant role in determining the ability of hydrocarbons to act as initiators for skin tumor induction when administered systemically.

The permanent nature of the initiating action of urethane was clearly shown by the experiments in which the promoting treatment with CR was delayed for 246 days. In these experiments similar results were obtained with both intraperitoneal and subcutaneous injections of urethane. The incidence of papillomas was compared at 210 days after the beginning of promoting treatment in the groups where promotion was delayed for 14 days and for 246 days. The period 210 days was selected because after this time interval papillomas coalesce and are difficult to score; furthermore, in the experiment in which the interval was 246 days, animal mortality was increasing. The data at 210 days after the beginning of promotion are not reflected in the tables and are as follows: in the group given subcutaneous injection of urethane, there were 12 tumors in 7 animals in the delayed promotion group and 5 tumors in 5 animals in the group in which the interval was 14 days. Similarly, following intraperitoneal injection of urethane, comparable tumor yields were 18 tumors in 8 animals with delayed promotion and 10 tumors in 6 animals with the usual interval of 14 days. Although the tumor yields with delayed promotion appear to be larger than in experiments with a 14 day interval between initiation and promotion, a statistical evaluation ($\chi^2$) of the data from these small test groups showed no significant differences between the two series.

On the other hand, in the experiment with urethane initiation and CR promotion, the latent period, *i.e.*, the interval between the beginning of promoting treatment and first appearance of papillomas, was substantially shorter in groups where the promoting treatment was started after an 8 month interval. The times to first tumors, 32 and 42 days (Tables I and II), in the delayed promotion experiments, are similar to those observed for skin initiation with DMBA followed by promotion with CR. Longer latent periods, 63 and 97 days respectively, were found in the groups where promoting treatment was started 14 days after systemic initiation.

The experiments described here indicate that for the bioassay of potential promoting agents, skin application of the initiating agent is preferable to systemic initiation, both in terms of time to first tumor and final tumor yields.

SUMMARY

A study was made of the promoting activity of phorbol esters from *Croton tiglium* L., tobacco leaf extract and cigarette tar following systemic initiation with DMBA, urethane or BP given by a single subcutaneous or intraperitoneal injection.

DMBA was shown to be a potent systemic initiator at doses even as low as 5 $\mu$g., while BP at 500 $\mu$g. was ineffective as a systemic initiator for skin tumors.

The permanent nature of such systemic initiation was shown in experiments in which skin tumors were induced when promoting treatment was delayed for 246 days. In these experiments there was no diminution in tumor yield and the latent period was shorter when compared to experiments in which the interval was 14 days.

WTE and WCT were active tumor-promoting agents on mouse skin when the initiating agent was given systemically. The latent period, however, was longer and the tumor yields lower than in corresponding experiments in which CR was used as a promoter.

For the purpose of bioassay of potential promoting agents, skin application of the initiator is preferable to systemic initiation.

This work was supported by Contract PH43–64–938, grants CA–06989 from the National Cancer Institute of the National Institutes of Health and No. ES–0014 from the Bureau of State Services, U.S. Public Health Service.

### REFERENCES

Berenblum, I. and Haran-Ghera, N.—(1957) *Br. J. Cancer*, **11**, 85.
Conney, A. H., Miller, E. C. and Miller, J. A.—(1957) *J. biol. Chem.*, **228**, 753.
Hieger, I.—(1965) *Br. J. Cancer*, **19**, 761.
Poel, W. E.—(1963) *Natn. Cancer Inst. Monogr.*, No. 10, 611.
Van Duuren, B. L. and Orris, L.—(1965) *Cancer Res.*, **25**, 1871.
Van Duuren, B. L., Sivak, A., Segal, A., Orris, L. and Langseth, L.—(1966) *J. natn. Cancer Inst.*, **37**, 519.